# A Preliminary Survey of Species Composition of Termites (Insecta: Isoptera) in Samunsam Wildlife Sanctuary, Sarawak

**DOI:** 10.21315/tlsr2017.28.2.15

**Published:** 2017-07-31

**Authors:** Norsyarizan Jamil, Wan Nurainie Wan Ismail, Siti Shamimi Abidin, Mazdan Ali Amaran, Ratnawati Hazali

**Affiliations:** Department of Zoology, Faculty of Resource Science and Technology, Universiti Malaysia Sarawak, 94300 Kota Samarahan, Sarawak, Malaysia

**Keywords:** Samunsam Wildlife Sanctuary, Termite, Composition, Termitinae

## Abstract

A survey on termite species composition was conducted in Samunsam Wildlife Sanctuary, Sarawak in February 2015. Overall 19 species of termite belonging to 13 genera and 8 subfamilies was found in the sanctuary. It was recorded the subfamily of Termitinae had the highest number of species (6 species, equal to 31.58% of total species), followed by Nasutermitinae (3 species, 15.79%), Macrotermitinae, Amitermitinae, Rhinotermitinae, Coptotermitinae, (2 species, 10.53% respectively), and Heterotermitinae, Termitogetoninae (1 species, 5.26% respectively). Since this rapid survey is the first termite assemblage representation in Samunsam Wildlife Sanctuary, the preliminary result may serve as the baseline data for termite composition in the area. Therefore, a whole coverage for the area within this sanctuary would definitely increase the number of termite species found in the sanctuary.

## Abstract (ru)

Kajian mengenai komposisi spesis anai-anai telah dijalankan di Pusat Perlindungan Hidupan Liar Samunsam, Sarawak pada 2015. Penilaian ini telah dijalankan bermula dari 7 sehingga 13 Februari 2015. Hasil kajian telah menemukan 19 spesis yang terdiri daripada 19 genera dan 8 subkeluarga di Pusat Hidupan Liar Samunsam. Subkeluarga Termitinae telah direkodkan mempunyai jumlah spesis yang tertinggi (6 spesis, bersamaan 31.58% daripada jumlah spesis), diikuti oleh Nasutermitinae (3 spesis, 15.79%), Macrotermitinae, Amitermitinae, Rhinotermitinae, Coptotermitinae (2 spesis, 10.53% masing-masing), dan Heterotermitinae, Termitogetoninae (1 spesis, 5.26% masing-masing). Kajian yang lengkap dan tempoh masa yang lebih panjang di kawasan ini akan pasti dapat menemukan lebih banyak komposisi spesis di pusat perlindungan hidupan liar ini.

Termites are one of the most popular insects that have been studied nowadays due to its capability to cause high economic damages (Bong *et al.* 2012). They are reported to destroy building and premises, become agricultural pests such as oil palm plantation, garden landscapes (Lee 2002) and bring damages to any cellulose materials such as books, papers, blanket, windows frame, furniture and man-made fabrics (Chao *et al.* 1989; Ibrahim & Adebote 2012). Although termites are known as destructive pests in building premises of urban areas (Van Quang *et al*. 2014) and have potential to affect economic growth, they are the most dominant inhabitants in tropical and subtropical regions, yet act as vital decomposer of cellulose materials in forest ecosystem (Pearce 2006; Vaessen *et al.* 2011).

Tho (1992) listed an estimated number of 175 species of termites from 42 genera and three families (Kalotermitidae, Rhinotermitidae and Termitidae) all across Peninsular Malaysia. A more recent review on termite taxonomy in the Sundaland region by Gathorne-Hardy (2004) listed about 132 species of termites in the Peninsula. There are about 104 species comprising 33 genera have been recorded in Sabah (Thapa 1981). Being responsive to habitat disturbance and environmental changes, the termite composition and assemblages in an area may be used as a model to evaluate the disturbance effects on an ecosystem (Davies 2002) and as potential indicator to investigate climatic change in a given area (Gathorne-Hardy *et al*. 2002). The study was conducted to investigate the composition of termites in the Samunsam Wildlife Sanctuary, the protected area in Sarawak. It is important to have faunal checklist of the area. The assessment will partially fill the gap of knowledge on the distribution of termites in Sarawak and serve as baseline data for future research.

Samunsam Wildlife Sanctuary is located at western tip of Sarawak (1° 55.00′ N 109° 36.00 E) (Fig. 1). Gazetted in 1979, Samunsam Wildlife Sanctuary is the first wildlife sanctuary to be established in Sarawak (Hazebroek & Abang Kashim 2000). The main access to the park is by a 60 min barge ride from Sematan jetty. Samunsam Wildlife Sanctuary was gazetted as a protected area mainly to protect the endemic proboscis monkey (*Nasalis larvatus*), Muller’s Bornean gibbons (*Hylobates muelleri*), silvered langurs (*Presbytis cristata*) and other wildlife species. The sanctuary which covers 6090 ha consists of mangrove, riverine, kerangas and mixed dipterocarp forests (Bennett & Sebastian 1988; Hazebroek & Abang Kashim 2000). The diverse vegetation exist in the sanctuary can supports such unique fauna by providing wide varieties of food sources and niches.

Survey was carried out along the natural trail (Fig. 1) and the sampling method is based on protocol established by Jones and Eggleton (2000). The 100 m belt transect consisted of 20 plots of 5 × 2 m^2^ that were sampled sequentially. Each plot was sampled for 30 min by two workers and collection of samples were made in the following microsites: forest litter, dead wood, tree trunks and buttress roots, mounds, soil surface to 5 cm depth (10–15 points) and runways to 2 m height in the vegetation. The survey was conducted for seven consecutive days from 7 to 13 February 2015. Termites were surveyed twice daily in the morning from 0900 to 1200 and 1400 to 1700, focussing on the termite nests, forest floor and dead woods. Approximately a total of 2 km of trail was covered representing different habitats including mangrove forest, swamp forest, mixed dipterocarp forest, riverine forest, and kerangas forest.

About 10 to 20 termite soldiers were collected from each colony found during survey. The termite specimens were collected and kept in small vials with 70%–80% ethanol. The termite species identification was made in the Parasitology Laboratory, Universiti Malaysia Sarawak following Tho (1992), Thapa (1981), Gathorne-Hardy (2004), Syaukani and Thompson (2011) and other relevant publications.

The photographs of the soldiers were taken using a Motic SMZ-16B Series stereomicroscope attached to a Moticam 2000 camera and then the image sequences were combined with Helicon Focus 6 software. Calibrated measurements were taken by using Motic Image Plus 2.0 software. The soldier identification features include (1) shape and characteristics of the head and mandibles, (2) antennae, (3) post-mentum, (4) pro-, meso- and meta-notum, (5) size and colouration of termites, and (6) other individual characteristics.

A total of 17 species identified were belong to two families, eight subfamilies and twelve genera (Table 1). Termites found were assigned into one of five feeding groups based on classification given in Collins (1984) and Eggleton *et al.* (1997) whether (i) wood feeders (termites feeding on living or dead wood), (ii) wood-litter feeders (foraging termites feeding on leaf litter or woody litter), (iii) soil feeders (termites feeding on humus and mineral soil), (iv) soil-wood interface feeders (termites feeding on highly decayed, friable and soil-like wood), or (v) epiphyte feeders (termites feeding on lichens, epiphytes and other free living non-vascular plants).

From a total of 17 species collected (Table 1), 11 species were assigned as wood feeding (W) species and by far the most common in all transects, represented by genera of *Nasutitermes, Havilanditermes, Prohamitermes, Microcerotermes, Schedorhinotermes, Coptotermes, Heterotermes* and *Termitogen.* Four species of soil humus feeders (S/H) dominated by *Discuspiditermes*, *Procapritermes* and *Pericapritermes*. Wood-litter feeders (WL) was found to be the least feeding group in the area represented by *Odontotermes mathuri* and *O. dentriculatus* (Table 1). Only the soil wood and epiphyte feeder were not encountered in the present study. Figure 2 represented the comparison between percentage of termite species and feeding group. *S. brevialatus* dominated wood feeders (n = 19) by 21.1% followed by *C. sepangensis* (15.8%), *C. curvignathus, N. matangensiformis* and *S. javanicus* with 10.5% respectively. For both wood-litter feeders (WL) and soil/humus feeders (S/H), they are equally represented, 50% by *O. mathuri* and *O. dentriculatus* respectively for WL group and 25% by *D. nemorosus*, *D. paramkhamensis*, *P. latignathus* and *P. neosetiger* respectively for S/H group. Data observed were further analysed for Chi Square test by using statistical computation program SPSS 18.0. The comparison between (i) the number of species and feeding group and (ii) the number of species and subfamily were computed to their significance.

Table 2 shows the percentage of species number encountered in Samunsam Wildlife Sanctuary. The subfamily Termitinae had the highest number of species (4 species, equal to 23.53% of total species), followed by Nasutermitinae (3 species, 17.65%), Macrotermitinae, Amitermitinae, Rhinotermitinae, Coptotermitinae, (2 species, 11.76% respectively), and Heterotermitinae, Termitogetoninae (1 species, 5.88% respectively). Two species of genus *Coptotermes* were found in this study, *C. curvignathus* (Fig. 3) and *C. sepangensis (*Fig. 4)*.* The *Coptotermes* is an important genus of subterranean termites as structural pests in Malaysia with five species; *C. curvignathus* (Holmgren), *C. travians (*Haviland), *C. kalshoveni (*Kemner), *C. sepangensis (*Krishna) and *C. havilandi (*Holmgren) (Lee 2002).

A study by Hanis *et al.* (2014) and Vaessen *et al.* (2011) had recorded the family of Termitidae as the dominant family found and the largest subfamily was Nasutitermitinae. In the present study, family of Termitidae was found the dominant family as well, yet the largest subfamily was Termitinae. Two genus of *Nasutitermes* (Fig. 5) and *Havilanditermes* (Fig. 6) under Nasutitermitinae family was only encountered, which is poorly represented in this area compared to Hanis *et al.* (2014) and Vaessen *et al.* (2011) data. The *Scheidorhinotermes* spp. (Fig. 7) of Rhinotermitinae subfamily was recorded to have the most total number of hits in Rhinotermitidae family (Table 1).

Further statistical analysis of Chi-square showed that the *p*-values of each data observed is smaller than α = 0.05 (Table 3). This indicated both termite feeding groups and subfamilies are significantly different with the termite species as shown in Table 3.

The wood feeding species dominated in the area by relative abundance of 19 total species hits (Table 1) explained the characteristics of the Samunsam Wildlife Sanctuary which full of tree trunks with humid soil conditions. Most of wood feeder termites only feed on dead trunks and have less contact with soil while certain are infesting on a living tree. The wood feeding group hold the ability to hollow out wood from within, without penetrating the surface. The least disturbance caused by human activity often resulting in increasing termite species richness, abundance and function by providing more structural and physical complexity which provides microhabitats for termites (Jones *et al.* 2003).

The lower percentage of soil humus feeding termite may be due to the location of the study sites which were in close proximity of water bodies (river, streams and lake). Soil feeders are said to account for more than 60% of the known termite species (Eggleton 2000). However, only three genera of *Discupiditermes, Procapritermes,* and *Pericapritermes* (Fig. 8) with four species encountered in this study (*D. paramkhamensis, D. nemorosus, P. neosetiger* and *P. latignathus*). The sampling period was done after high annual rainfall, and certain parts of the study areas may be flooded or inundated during the time. The occasional soil inundations may affect termite’s habitat and create unfavourable condition to soil termites especially the soil feeder which probably explain their low composition in this study. As reported by Collins (1979), high annual rainfall and seasonal flooding create difficulties in termites nesting and foraging. Dibog *et al.* (2000) also observed a significant effect of seasonal rainfall on termite diversity and abundance that decline with increasing rainfall.

Wood leaf feeding species is the termite that forages for leaf litter and small woody items litter in various stages of decay. This group includes some subterranean and other mound-building Macrotermitinae (with fungal association) (Eggleton *et al.* 1996). In this study, only two mounds of Macrotermitinae species were found along transect in the study location. According to Wood and Johnson (1986), *Odontotermes* are known to build huge mounds of selected clay-rich subsoil. Commonly, the mound builder species are more prevalent in post clearing land-spaces such as in oil palm plantation area or along the side road. The least species composition of mound builder species such as *Odontotermes* may due to the area covered in the study area. This supported by Axelson and Anderson (2012) who suggested that the mounds predominantly occurred in sites with denser canopies. Canopy cover can protect the mounds from sun exposure, soil erosion due to heavy rainfall, provide microclimate conditions and ground moisture. It can be suggested that if the sampling period was prolonged and more are were covered, the chances of more termite mound builder can be found in the study location as the location are very suitable for mound builder species of termites with dense canopies and have high moisture.

The overall species found in Samunsam Wildlife Sanctuary was relatively low compared to the number of species found in other forests areas and peat areas reported from several studies conducted in Borneo (e.g., Eggleton *et al*. 1997, Jones & Prasetyo 2002, Vaessen *et al*. 2011). A study of termite assemblages along a land use gradient on peat areas in Sarawak (Vaessen *et al*. 2011) had recorded the overall 20 species from four different sites which relatively higher than Samunsam Wildlife Sanctuary. However, the total number of species found in near natural peat swamp forest in their study was relatively lower than our present study with recorded to have 11 species found and relative abundance of 16 termite hit species. As our present study was not designed to determine the effect of different gradient of land use, no conclusion can be drawn from our result.

It was expected that more termite species from Samusam Wildlife Sanctuary would found because the area is protected from any logging activities since the establishment in 1979. Back then during 1969, logging activities had been done extensively and intensively may disturb the composition of the termite especially with soil feeder termite being the most at risk to the loss of primary forest. Contrary to soil feeder termite, wood feeder termite can survive in disturb area and in some cases have better survivorship in disturb area that provide greater organic output. This shown that different feeding group of termites would give different effects to habitat disturbances as explained by previous studies (Eggleton *et al*. 1997, Eggleton & Tayasu, 2001).

Conclusively from this preliminary study, Samunsam Wildlife Sanctuary harbours a minimum of 17 species of termites. This study is the first termite assemblage survey at Samunsam Wildlife Sanctuary and the results can be used as the baseline data for future survey. More extensive explorations and inventories are suggested in order to get a better representation of the entire termite composition of this area which will further relate to the species response to landscape changes in Western Sarawak.

## Figures and Tables

**Figure 1:**
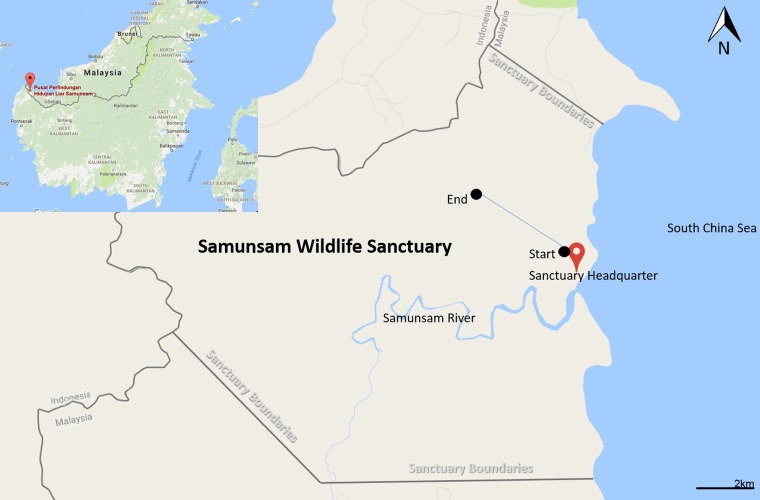
Samunsam Wildlife Sanctuary location.

**Figure 2:**
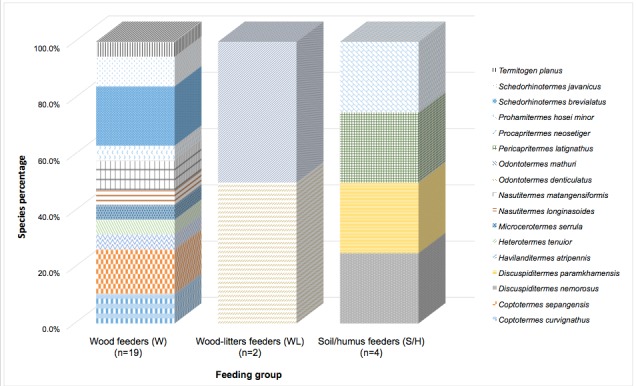
Comparison between percentage of termite species and feeding group.

**Figure 3:**
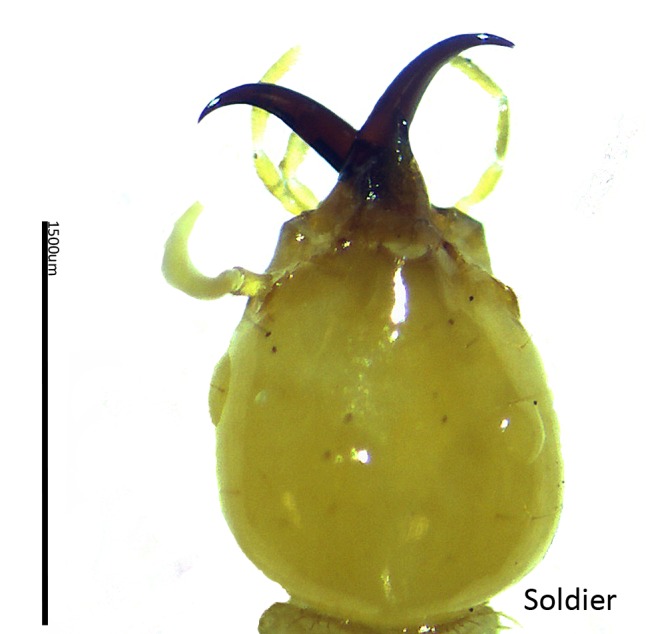
The soldier head of *C. curvignathus*

**Figure 4:**
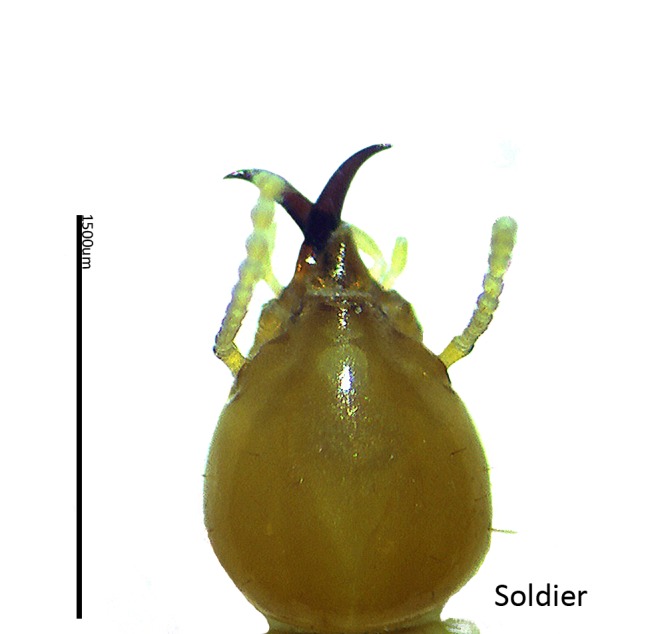
The soldier head of *C. sepangensis*

**Figure 5:**
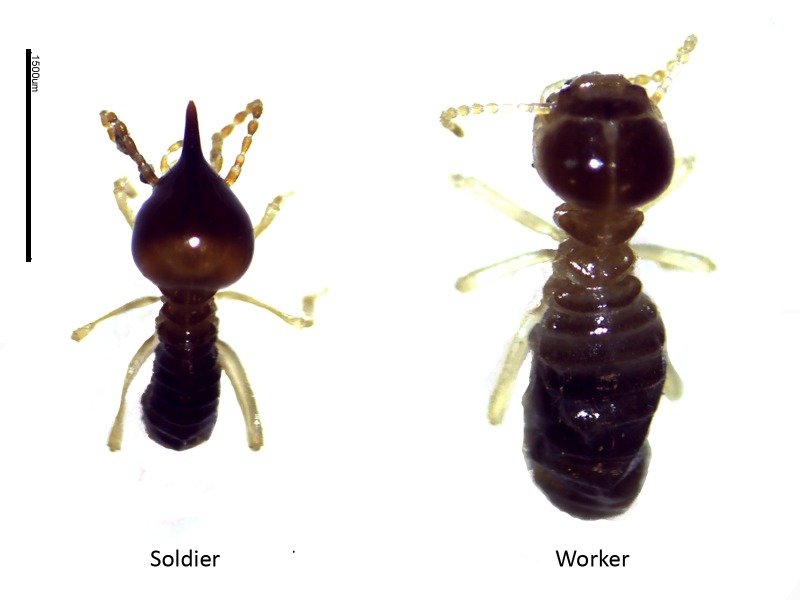
Soldier and worker *Nasutitermes*

**Figure 6:**
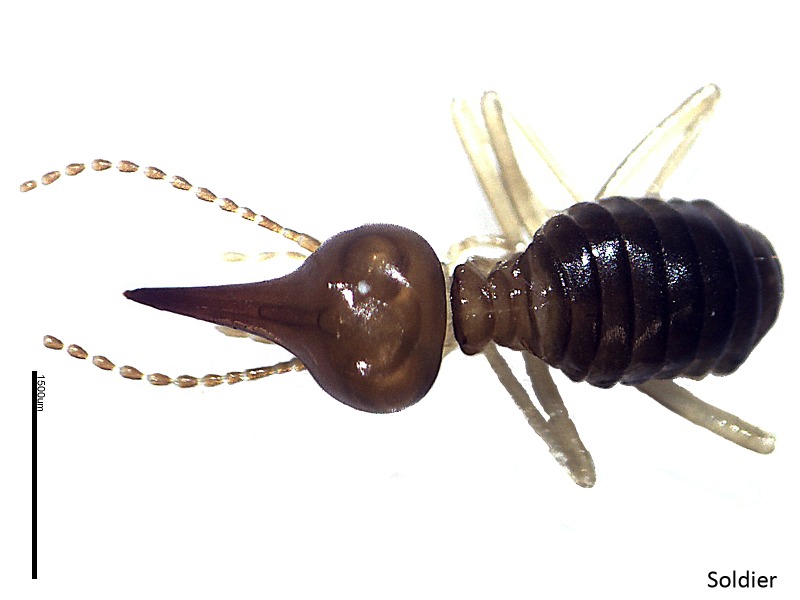
Soldier of *Havilanditermes*

**Figure 7:**
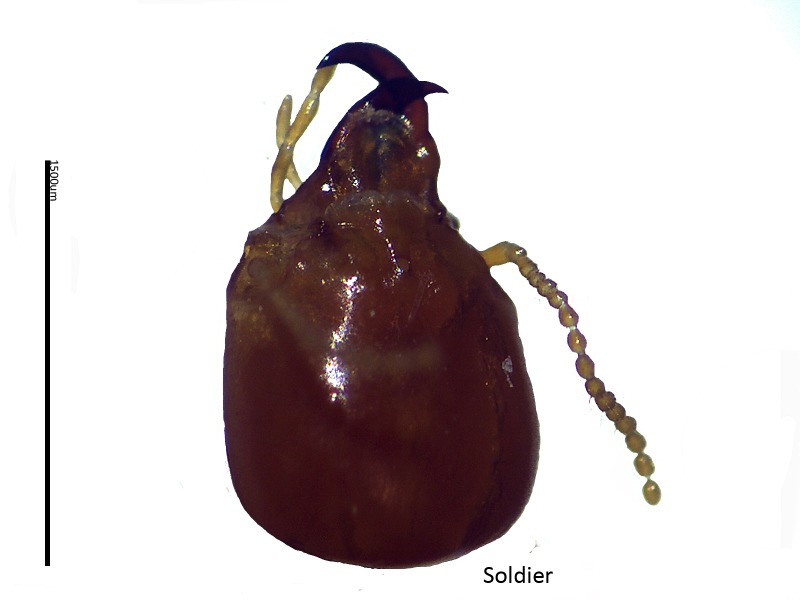
Soldier head of *Scheidorhinotermes* spp.

**Figure 8:**
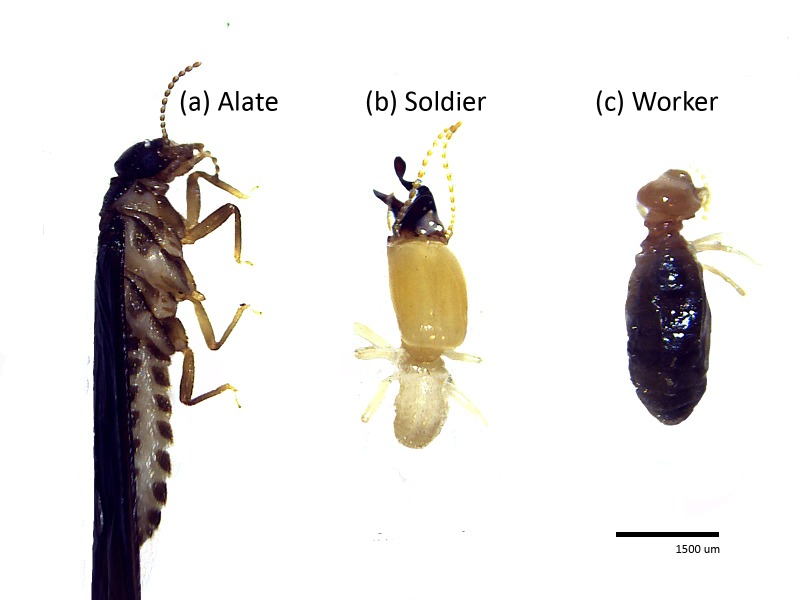
Alate (a), soldier, (b) and worker (c) *Pericapritermes* spp.

**Table 1:** Relative abundance of termite species collected in Samunsam Wildlife Sanctuary, based on number of ‘hits’ of each species in a transect (the presence of species in one section represents one hits). Feeding group: W= wood feeders; WL = wood-litters feeders; S/H = soil/humus feeders.

Family	Subfamily	Species	Feeding group	Relative abundance of species
Termitidae	Macrotermitinae	*Odontotermes mathuri*	WL	1
		Odontotermes denticulatus	WL	1
	Termitinae	*Discuspiditermes paramkhamensis*	S/H	1
		*Discuspiditermes nemorosus*	S/H	1
		*Procapritermes neosetiger*	S/H	1
		*Pericapritermes latignathus*	S/H	1
	Nasutitermitinae	*Nasutitermes matangensiformis*	W	2
		*Nasutitermes longinasoides*	W	1
		*Havilanditermes atripennis*	W	1
	Amitermitinae	*Prohamitermes hosei minor*	W	1
		*Microcerotermes serrula*	W	1
Rhinotermitidae	Rhinotermitinae	*Schedorhinotermes brevialatus*	W	4
		*Schedorhinotermes javanicus*	W	2
	Coptotermitinae	*Coptotermes curvignathus*	W	2
		*Coptotermes sepangensis*	W	3
	Heterotermitinae	*Heterotermes tenuior*	W	1
	Termitogetoninae	*Termitogen planus*	W	1

Total number of species				17

Relative abundance (total hits)				25

**Table 2:** The genera composition of termites in Samunsam Wildlife Sanctuary.

No.	Scientific name	Species number	Percentage (%)
	RHINOTERMITIDAE		
	Rhinotermitinae Froggatt	2	11.76
1	*Schedorhinotermes* Silvestri	2	
	Coptotermitinae Holmgren	2	11.76
2	*Coptotermes* Wasmann	2	
	Heterotermitinae Froggat	1	5.88
3	*Heterotermes* Froggatt	1	
	Termitogetoninae Holmgren	1	5.88
4	*Termitogen* Desneux	1	
	TERMITIDAE		
	Macrotermitinae Kemner	2	11.76
5	*Odontotermes* Holmgren	2	
	Termitinae Sjostedt	4	23.53
6	*Discupiditermes* Krishna	2	
7	*Procapritermes* Holmgren	1	
8	*Pericapritermes* Silvestri	1	
	Nasutitermitinae Hare	3	17.65
9	*Nasutitermes* Dudley	2	
10	*Havilanditermes* Light	1	
	Amitermitinae Kemner	2	11.76
11	*Prohamitermes* Holmgren	1	
12	*Microcerotermes* Silvestri	1	

	Relative Abundance	17	100

**Table 3:** Statistical analysis of Chi-square obtained for H_1_: Species vs Feeding groups and H_2_: Species vs Subfamilies

Hypothesis testing	Significance value
H_1_: Species vs Feeding groups	*p* = 0.022 < α = 0.05*
H_2_: Species vs Subfamilies	*p* = 0.000 < α = 0.05*

Chi-square test α = 0.05;

* = significantly different
